# Recombinant SARS-CoV-2 spike S1-Fc fusion protein induced high levels of neutralizing responses in nonhuman primates

**DOI:** 10.1016/j.vaccine.2020.06.066

**Published:** 2020-07-31

**Authors:** Wenlin Ren, Hunter Sun, George F. Gao, Jianxin Chen, Sean Sun, Rongqing Zhao, Guang Gao, Yalin Hu, Gan Zhao, Yuxin Chen, Xia Jin, Feng Fang, Jinggong Chen, Qi Wang, Sitao Gong, Wen Gao, Yufei Sun, Junchi Su, Ailiang He, Xin Cheng, Min Li, Chenxi Xia, Maohua Li, Le Sun

**Affiliations:** a AnyGo Technology Co., LTD, D1117 Xinhua International Plaza, 89 Dayangfan Rd, Beijing, China; b AbMax Biotechnology Co., LTD, 99 Kechuang 14th St., BDA, Beijing 101111, China; c Institute of Microbiology Chinese Academy of Sciences, China; d ZhenGe Biotechnology Co., LTD, Shanghai, China; e SinoVac, Beijing, China; f Advaccine (Suzhou) Biopharmaceuticals, Co., LTD, Suzhou, Jiangsu, China; g Dept. Lab Medine, Nanjing Univ. Medical School, Nanjing, Jiangsu 210008, China; h College of Public Health, Fudan Univ., Shanghai, China

**Keywords:** COVID-19, SARS-CoV-2, Spike protein, Vaccine

## Abstract

The COVID-19 outbreak has become a global pandemic responsible for over 2,000,000 confirmed cases and over 126,000 deaths worldwide. In this study, we examined the immunogenicity of CHO-expressed recombinant SARS-CoV-2 S1-Fc fusion protein in mice, rabbits, and monkeys as a potential candidate for a COVID-19 vaccine. We demonstrate that the S1-Fc fusion protein is extremely immunogenic, as evidenced by strong antibody titers observed by day 7. Strong virus neutralizing activity was observed on day 14 in rabbits immunized with the S1-Fc fusion protein using a pseudovirus neutralization assay. Most importantly, in <20 days and three injections of the S1-Fc fusion protein, two monkeys developed higher virus neutralizing titers than a recovered COVID-19 patient in a live SARS-CoV-2 infection assay. Our data strongly suggests that the CHO-expressed SARS-CoV-2 S1-Fc recombinant protein could be a strong candidate for vaccine development against COVID-19.

## Highlights

• CHO-expressed S1-Fc protein is very immunogenic in various animals and can rapidly induce strong antibody production.
• S1-Fc protein solicits strong neutralizing activities against live virus.
• Stable CHO cell line expressing 50 mg/L of S1-Fc, making it an accessible and affordable option for worldwide vaccination.

## 1 Introduction

The SARS-CoV-2 was first identified in Wuhan, China at the end of 2019 [1], [2], [3], [4], [5]. In only five months, the virus has caused a global pandemic, with over 7,700,000 confirmed cases and over 426,000 deaths worldwide. As a novel coronavirus with no effective treatments or drugs currently available, a vaccine is in dire need of development. Several broad approaches to the development of a COVID-19 vaccine have emerged, including DNA vaccines, RNA vaccines, viral vector vaccines, recombinant subunit vaccines, and dead viral preparations [6]. Among these, an RNA vaccine from Moderna was the first to reach human trials in early March in the US, followed by Cansino’s adenoviral vector vaccine which began human trials in China later in the same month.

Considering that the spike protein is the receptor-binding protein that mediates viral-cell fusion during the initial infection event [7], [8], it has been identified as a primary target for vaccine design. The spike protein has a total of 1273 amino acids, which can be divided into two major domains according to their structures and functions [7], [9]. The first half is the S1 protein, which contains the receptor binding domain (RBD) sequence and is located at the N-terminus of the spike protein [7], [10], [11]. The second half is the S2, serving as a trimeric structure that supports the S1′s RBD and has a fusion bundle which protrudes out into the host cell’s membrane after it is triggered by the S1 coming into contact with ACE 2 [9], [11], [12]. Due to the fact that most of the neutralizing epitopes are located within the S1 region, proteins containing the RBD, full-length of S1, full-length of S (S1 + S2) or even a trimeric S approach have been considered as candidates for vaccine development [13].

In this study, we fused the full length SARS-CoV-2 S1 protein (GenBank: QIC53204.1, Gln14-Arg685) with the Fc region of human IgG1 (GenBank: CAR58103.1, Glu98-Lys329) as our vaccine candidate and expressed the recombinant protein using a stable CHO-K1 cell line. The purified S1-Fc protein was formulated with different adjuvants and used to immunize different species of animals, such as mice, rabbits, and macaques. Besides eliciting high levels of anti-S1 antibodies in all tested animals, high neutralizing activities against SARS-CoV-2 were also found in the anti-sera from macaques. These results indicate that the S1-Fc fusion protein can effectively induce humoral immune responses in various animals and can elicit high levels of neutralizing antibodies in macaques.

## 2 Materials and methods

### 2.1 Materials

AD11.10 (saponin based microemulsion) and AD20Gold+ (nanoemulsion with synthetic MPL) adjuvants were from Advaccine, China. Freund's complete adjuvant (CFA) was purchased from SIGMA, USA. Female BALB/c mice were obtained from Vital River Co., China. New Zealand White rabbits were purchased and hosted at Longan Co., China. Macaques were generated and hosted at Xieerxin Biotech., China. The mice were group housed, while the rabbits and macaques were caged separately. All the animals were fed with general diet, while the vegetables and fruits were added additionally for macaques. Drinking waters for all animals were purified and autoclaved. Peroxidase conjugated secondary antibodies were sourced from Jackson Immunoresearch, USA. CHO-expressed SARS-CoV-2 S1-Fc fusion protein and S1-6 × His were produced by ZhenGe Biotech., China.

### 2.2 Immunizations

4 weeks-old female BALB/c mice, 12–15 weeks-old female NZW rabbits and 3 ~ 4 years old Macaques were immunized with CHO-expressed SARS-CoV-2 S1-Fc fusion after formulated with adjuvants according to manufacturer’s instructions. Briefly, 4-weeks old female BALB/c mice were immunized with S1-Fc protein immersed in AD20Gold^+^ (9.2 μg on Day 0, 3, 7 and reduced to 0.575 μg on Day 9 and 11 intramuscularly). NZW rabbits were also immunized with S1-Fc protein immersed in AD20Gold^+^ (100 μg on Day 0, 4, 7 and reduced to 50 μg on Day 11, 14 and 18 intramuscularly). For immunization of macaques, CFA was used to prime the primate at the first injection and AD11.10 was used to boost them (250 μg in CFA on Day 0 subcutaneously, and 250 μg in AD11.10 on Day 4, 9, 22 and 26 intramuscularly). The immunization process is shown in Table S1. Blood samples were collected at different time points for measurement of antibody levels and neutralizing titers. All protocols involved the use of animals were approved by the IACUC/Ethical Committee/ Animal Welfare.

### 2.3 Enzyme-linked immunoassay

The assay was carried out as described by Zhao RQ, et al [14]. Briefly, wells of 96-well plate were coated with 1.5 μg/mL of SARS-CoV-2 S1-6 × His protein in PBS, blocked with 3% BSA-PBS. Serial dilutions of sera were first diluted with the normal sera from the same species, further diluted with Sample Dilution Buffer (20% Calf serum in PBS, 20%CS-PBS). 100 μL of the diluted samples were transferred to each well of the plates. The captured antibodies were probed with Horseradish-Peroxidase (HRP)-conjugated secondary antibodies. For example, to detect monkey anti-S1 total antibodies, HRP-conjugated AffiniPure Goat Anti-Human IgG (H + L) from Jackson Immuno Research (Catalogue Number: 109–035-003) was used. For IgM detection, HRP-labeled Monoclonal Mouse Anti-Human IgM (μ) from Cellway-lab (Catalogue Number: C030201) was used. After final washes, HRP substrate TMB solution (Beijing Kwinbon, China) was added and absorbencies were measured at 450 nm with a microplate reader.

### 2.4 Pseudovirus neutralization assay

HEK 293 T cells and ACE2-overexpressed HEK 293 T cells (ACE2-293 T) were cultured in DMEM (Gibco, USA) supplemented with 10% heat-inactivated fetal bovine serum (FBS, Gibco), 50 U/ml penicillin (Gibco) and 50 μg/ml streptomycin (Gibco) at 37 °C with 5% CO_2_. HEK 293 T cells were co-transfected with 10 μg of a plasmid encoding SARS-CoV-2 S protein and 10 μg of an env-deficient, luciferase-expressing HIV vector (pNL4–3.luc.RE) using Lipofectamine 2000 reagents (Life Technologies, USA). After 48 h, pseudovirus-containing culture supernatants were collected, filtered (0.45 μm), and stored at −80 °C in 1 ml aliquots. The 50% tissue culture infectious dose (TCID_50_) of a single thawed aliquot of pseudoviruses was determined in ACE2-293 T cells. For TCID_50_ measurement, serial dilutions of pseudoviruses were added to ACE2-293 T cells pre-seeded in 96-well plates. After 12 h incubation, the medium was replaced by DMEM containing 10% FBS, and cells were cultured for an additional 48 h. Cells were lysed with lysis buffer (Glo-lysis buffer, Promega), followed by addition of luciferase substrate (Bright-Glo luciferase assay substrate, Promega). Luciferase activity was measured using a GloMax 96 microplate luminometer (Promega) and wells producing relative luminescence units (RLU) above three times of the mean background value were defined as positive.

ACE2-293 T cells were seeded in 96-well plates at one day prior to infection. Serum samples were inactivated at 56 °C for 0.5 h, and then 3-fold serially diluted and incubated with 200 TCID_50_ of pseudoviruses for 1 h at 37 °C. The mixtures were then used to infect ACE2-293 T cells in triplicate. Following 12 h infection, wells were replenished with fresh medium and the luciferase activities of cells were determined 48 h later. Cells were lysed with lysis buffer (Glo-lysis buffer, Promega), followed by addition of luciferase substrate (Bright-Glo luciferase assay substrate, Promega). Luciferase activity was measured using a GloMax 96 microplate luminometer (Promega) and wells producing relative luminescence units (RLU) above three times of the mean background value were defined as positive. The 50% neutralization titer was calculated by probit analysis using the SPSS software.

### 2.5 SARS-CoV-2 neutralizing assay

Strain CN1 was chosen for neutralizing assay. SARS-CoV-2 virus titer was determined by microdose cytopathogenic efficiency (CPE) assay. Serial 10-fold dilutions of virus contained samples were mixed with 1–2 × 10^4^ Vero cells, and then plated in 96-well culture plate. After 3–7 days culture in a 5% CO_2_ incubator at 36.5 °C, cells were checked under a microscope for the presence of Cytopathic effect (CPE). Virus titer was calculated by the method of Karber [15].

Serum samples were inactivated at 56 °C for 0.5 h and serially diluted with cell culture medium in two-fold steps. The diluted sera were mixed with SARS-CoV-2 suspension of 100 TCID_50_ in 96-well plates at a ratio of 1:1, followed by 2 h incubation at 36.5 °C in a 5% CO_2_ incubator. 1–2 × 10^4^ Vero cells were then added to the serum-virus mixture, and the plates were incubated for 5 days at 36.5 °C in a 5% CO_2_ incubator. CPE of each well was recorded under microscopes, and the neutralizing titer was calculated by the dilution number of 50% protective condition.

## 3 Results

### 3.1 Production of SARS-CoV-2 S1-Fc fusion protein

The S1 subunit (14Q-685R) was fused with Fc fragment of human IgG1 genetically for ease of purification, constructed in pEE12.4, and expressed by a stable CHO cell line. S1-Fc fusion protein was purified from the culture supernatant using a Protein-A column, as shown in Fig. S1A. The purity of S1-Fc protein was evaluated by running SDS-PAGE gel under reduced and non-reduced conditions. A band between 130 and 180 kD was detected as monomer of the S1-Fc, while the dimer appeared as a single band well above 180 kD molecule weight marker (Fig. S1B).

### 3.2 Immunizations of animals with SARS-CoV-2 S1-Fc fusion protein

**Fig. 1:**
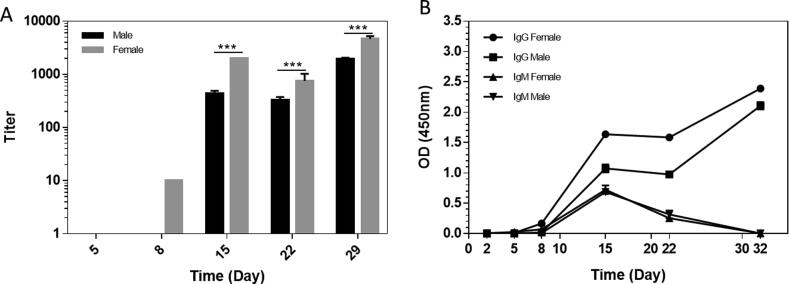
Anti-SARS-CoV-2 S1 antibody levels in S1-Fc immunized macaques. A) The ELISA titers of total anti-S1 antibodies in macaque sera. Sera were evaluated by ELISA using HRP-conjugated goat-anti-human IgG (H + L) secondary antibodies (P < 0.001, one-way Anova; n = 4). The horizontal coordinate is time and the vertical coordinate is the ELISA titer of anti-S1 antibodies in the sera. B) The levels of IgG and IgM in macaque sera. Sera were diluted 50 times with normal monkey serum and then diluted 20 times with sample dilution buffer. The samples were examined with either HRP-conjugated goat anti human IgG Fc-specific secondary antibodies (1:20000) or HRP-conjugated mouse monoclonal anti-μ-Chain of human IgM (1:5000). The horizontal coordinate is time and the vertical coordinate is absorbance value at 450 nm.

One female and one male macaque were immunized with S1-Fc protein with CFA and AD11.10. The sera were collected on different days (prior to, day 5, 8, 15, 22 and 32 post the first immunization) and evaluated by ELISA against SARS-CoV-2 S1-6 × His protein. The titers of total antibodies against SARS-CoV-2 S1 proteins were first examined by using HRP-conjugated goat anti-human IgG (H + L) secondary antibodies, which react with IgM, IgG1, IgG2, IgG3 and IgG4. As shown in Fig. 1A, on day 8, anti-S1 total antibodies were detected only in the female macaque. On day 15, good titers of anti-S1 total antibodies were observed in both macaques. However, on day 22, there was a 30% drop of total anti-SARS-CoV-2 S1 antibody titers in the female macaque. One more boost was given on Day 22 and the total antibody titers in both macaques increased greatly on Day 32.

Furthermore, we used HRP-labeled anti-human IgG Fc and HRP-conjugated mouse anti-human μ-Chain isotype-specific secondary antibodies to re-examine the levels of IgGs and IgM in the sera. As shown in Fig. 1B, only anti-S1 IgG antibodies were seen in the female macaque on day 8. On day 15, strong anti-S1 IgG titers were detected in both macaques, while the IgM levels were also detectable. Interestingly, after one more boost of S1-Fc on day 22, only a big increase in anti-S1 IgG titers on Day 32, meanwhile, no significant anti-S1 IgM titers was observed.

**Fig. 2:**
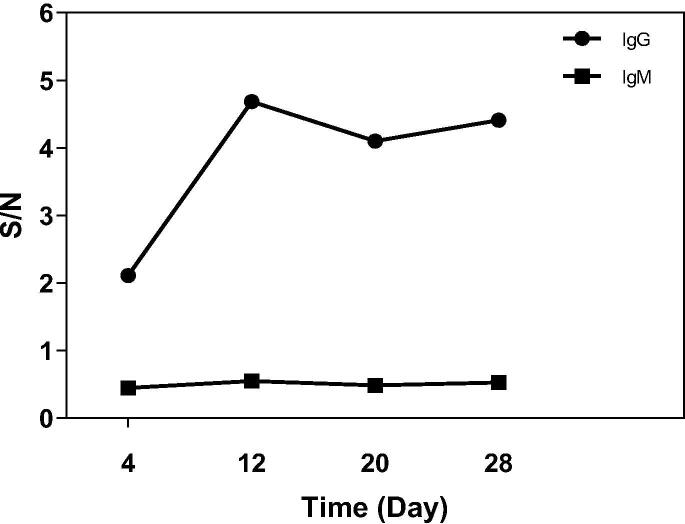
Levels of IgG and IgM in sera from COVID-19 convalescence patient. Sera were examined with either HRP-conjugated goat anti-human IgG Fc-specific secondary antibodies (1:20000) or HRP-conjugated mouse monoclonal anti-μ-Chain of human IgM (1:5000). The horizontal coordinate is time and the vertical coordinate is SNR (Signal to Noise Ratio).

Using the same ELISA assay, we examined the levels of anti-S1 IgG and IgM antibodies in the plasma samples collected at different days post onset of COVID-19 disease from the recovered patient. As shown in Fig. 2, the IgG antibodies can be detected 4 days after onset of disease and the titer increased on Day 12, but reduced significantly on Day 20. No IgM was detected.

The original purpose of this study was to generate positive control SARS-CoV-2 S1 antibodies for development of SARS-CoV-2 vaccine and serological assays. Some of the immunizations were done with the help of Freund's complete adjuvant (CFA), which is not suitable for human vaccination. However, high anti-S1 antibody titers were also observed in one female macaque which was immunized with 50 μg of S1-Fc immersed in 0.5 ml of AD10.11 after two IM on Day 0 and 10 (data not shown). To confirm this observation, later on, we switched the adjuvant from the harsh CFA to AD20Gold^+^ (nanoemulsion with synthetic MPL) which was designed for human use to study the potential of S1-Fc as the candidate for human COVID-19 vaccine.

**Fig. 3:**
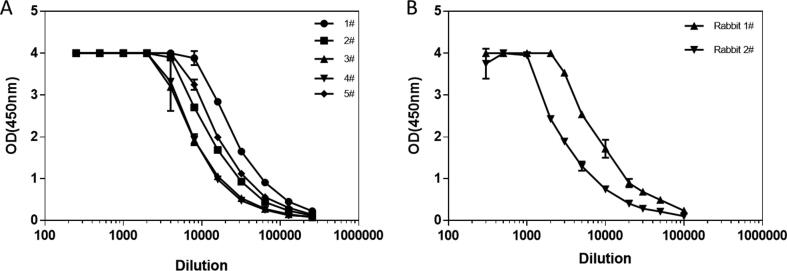
Anti-SARS-CoV-2 S1 antibody levels in S1-Fc immunized mice and rabbits. A) Serum samples collected from different mice were evaluated with S1-6 × His coated plates by ELISA using mouse IgG-specific secondary antibodies. The horizontal coordinate is dilution and the vertical coordinate is absorbance value at 450 nm. B) Serum samples collected from different rabbits were evaluated with S1-6 × His coated plates by ELISA using rabbit IgG-specific secondary antibodies. The horizontal coordinate is dilution and the vertical coordinate is absorbance value at 450 nm.

Five 4-weeks old female BALB/c mice were immunized with S1-Fc protein as described previously. The sera were collected on Day 38 and evaluated by ELISA against SARS-CoV-2 S1-6 × His protein using HRP-conjugated goat anti-mouse IgG Fc-specific secondary antibodies. As shown in Fig. 3A, all five mice developed strong anti-S1 IgG antibody titers (64,000 to 256,000).

Two rabbits were also immunized with S1-Fc protein immersed in AD20Gold^+^. On day 27, the bleeds were collected for examination of anti-S1 antibody titers. Similar to what been observed in mice, the S1-Fc immunized rabbits also developed very high binding titers (100,000 or higher) against the S1 protein (see Fig. 3B).

### 3.3 Neutralization activities of SARS-CoV-2 S1-Fc fusion protein antisera

The virus neutralizing activities of the S1-Fc immunized sera were examined using either pseudo-virus or live SARS-CoV-2.

**Fig. 4:**
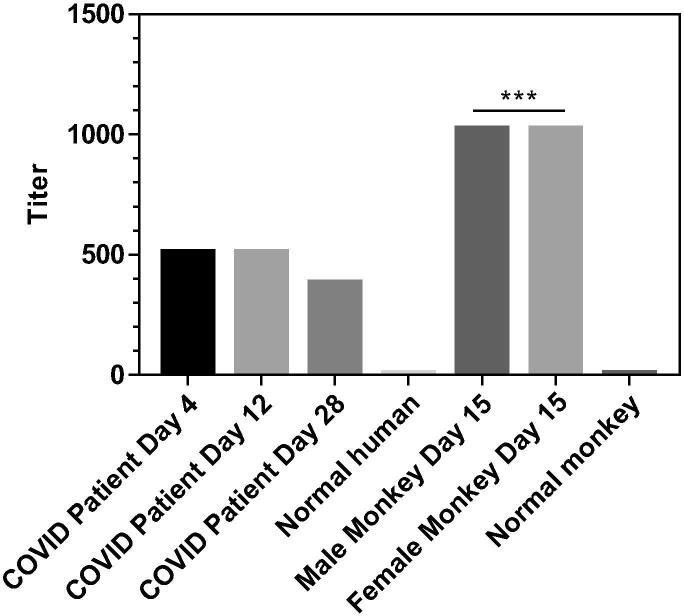
The neutralizing titers of S1-Fc-immunized macaque and COVID-19 Convalescent patient’s sera. The neutralizing titer of macaque and COVID-19 Convalescent patient’s sera was examined using live SARS-CoV-2 (P < 0.001, one-way Anova; n = 4). The vertical coordinate is the neutralizing titer.

We compared the neutralizing titers of the sera from S1-Fc immunized macaques with the ones of recovered COVID-19 patient’s plasma samples using live SARS-CoV-2. Heat-inactivated serum samples were pre-incubated with live SARS-CoV-2 suspension of 100 TCID_50_ for 2 h at 36.5 °C, then mixed with 1–2 × 10^4^ Vero cells, and co-culture for 5 days at 36.5 °C. Cytopathic effect (CPE) of each well was recorded under microscopes, and the neutralizing titer was calculated by the dilution number of 50% protection. As shown in Fig. 4, the virus neutralizing titers of human sera from the recovered COVID-19 patient were at 512 on Day 4 and Day 12, but dropped to 384 on Day 28 post the onset of disease, matched with the decrease of antibody levels in the plasma samples observed above on Day 20 and 28. Immunization of SARS-CoV-2 S1-Fc fusion protein with the help of CFA + AD11.10 as adjuvants induced very high neutralizing activities with titers > 1024 on Day 15 in both macaques against live SARS-CoV-2 infection.

**Fig. 5:**
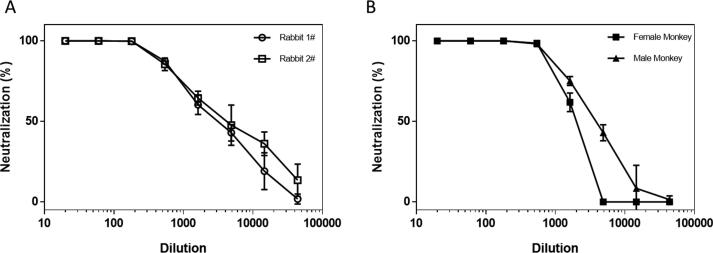
Pseudo-Virus neutralizing activities of antisera from S1-Fc-immunized rabbits and monkeys. A) The neutralizing activity of rabbit sera was evaluated with SARS-CoV-2 pseudo-virus. The horizontal coordinate is serum dilution and the vertical coordinate is the percentage of the neutralization. B) The neutralizing activity of macaque sera was evaluated with SARS-CoV-2 pseudo-virus. The horizontal coordinate is serum dilution and the vertical coordinate is the percentage of the neutralization.

The neutralizing activities of the sera from S1-Fc immunized rabbits or macaques were also examined using SARS-CoV-2 pseudo-virus. Briefly, heat-inactivated serum samples were pre-incubated with 200 TCID_50_ pseudo-viruses for 1 h at 37 °C. The mixtures were then used to infect ACE2-293 T cells. Three days later, the luciferase activities of the infected cells were measured. As shown in Table 1 and Fig. 5A, immunization of SARS-CoV-2 S1-Fc fusion protein with AD20Gold^+^ as adjuvant also induced very high neutralizing activities with IC50 titers > 3000 and IC90 titers around 440–501 in both rabbits on Day 27 after immunizations. As shown in Fig. 5B, excellent neutralizing titers also observed in sera from the two S1-Fc immunized macaques on Day 32. However, while the male macaque developed anti-SARS-CoV-2 S1 protein antibodies slower than the female one with lower binding titers, it surprisingly had much stronger neutralizing titer (3741) than the female macaque (1798) at the end.

**Table 1:** Pseudo-virus neutralizing titers of sera from S1-Fc immunized macaques and rabbits.

Sample	IC50	IC90
Rabbit serum 1# Day 27	3213	501
Rabbit serum 2# Day 27	4456	440
Normal macaque serum	76	<20
Female macaque serum Day 32	1798	824
Male macaque serum Day 32	3741	989

Our data clearly demonstrated that the CHO-expressed full length SARS-CoV-2 S1-Fc fusion protein can solicit very strong neutralizing activities against SARS-CoV-2 in a pretty short time period and could be a good candidate for COVID-19 vaccine development.

## 4 Discussion

The spike protein of the SARS-CoV-2 has a total of 1,273 amino acids and two major domains according to their structures and functions. The first half is an S1 protein which contains the RBD sequence and is located at the N-terminus of the spike protein [7]. The second half is the S2, serving as the base for the trimeric structure to support the S1 receptor binding structure in place. Since the RBD being located within the S1 region, we generated a S1-Fc fusion protein as a candidate for vaccine development.

Since the SARS-CoV-2 S1 protein is predicted to have at least 16 N-linked glycosylation sites [16], we chose mammalian cell CHO-K1 as the expression system to ensure the right glycosylation. CHO-K1 is also one of the most popular cell lines used to make human therapeutic antibody drugs.

Shringrix, a vaccine based on the recombinant gE protein of varicella-zoster virus, was launched in 2018 by GSK for prevention and treatment of shingles with great success [17]. The primary vaccination schedule consists of two dose of 50 μg/0.5 ml each at two month apart [17]. Assuming the same vaccination schedule for COVID-19, with the stable expression level at 50 mg/L of S1-Fc fusion protein, a 3,000 L CHO cell Bioreactor can easily produce 3 million dose of recombinant S1-Fc vaccine every two weeks. With the capacity of >1 million liters of mammalian cell biorectors in the world, our S1-Fc may be the only feasible way to make enough COVID-19 vaccines for the entire world within one year.

There are many therapeutic biological drugs such as TNF Receptor-Fc fusion protein (Enbrel) that have been used for treatments of AMD and other human diseases without any known side-effect caused by the Fc region of the drugs so it is safe to use the human IgG1′s Fc as the fusion partner for SARS-CoV-2 S1. Dimerization of S1-Fc fusion protein via the disulfide bonds formed within the hinge regions of Fc may also help to mimic the native 3D conformation of the binding pocket of oligomerized S protein *in vivo*. In addition, the human Fc domain fused to the C terminal of S1 protein greatly facilitates the purification of the recombinant protein following the standardized antibody drug manufacturing procedures.

Using the purified recombinant S1-Fc fusion protein as immunogen, we demonstrated it not only has very good immunogenicity in mice, rabbits and non-human primates, but also can elicit strong neutralizing activities against SARS-CoV-2 infection *in vitro*.

It was reported by Dr. F. Krammer’s group that SARS-CoV-2 infections selectively induced anti-SARS-CoV-2 S antibodies with IgG3 isotype [18]. Normally, IgG3 only consists 7% of the total IgGs in sera, while 60% is IgG1. However, in COVID-19 patients, over 60% of SARS-CoV-2 S antibodies were IgG3. IgG3′s half-life is only 7 days, the shortest one, while the other three IgG isotypes’ are 21-days long [19]. IgG3 is also the isotype with the strongest inflammatory activities. Together with the quick drop of SARS-CoV-2 S1 antibody titers observed both in human patient and the female macaque in this study, we started a new vaccine study with much smaller dose (1, 3 and 6 μg) and weaker adjuvant, hoping to induce less IgG3 isotype of anti-SARS-CoV2 antibodies.

We have been using the SARS-S1-6 × His protein to challenge the S1-Fc immunized mice which have developed strong anti-SARS-CoV-2 S1 titers, and have not observed any abnormality with the mice.

## 5 Conclusion

In this study, we established a CHO-K1 cell line that stably expresses the SARS-CoV-2 S1 protein with human IgG Fc fused to its C terminal. The purified recombinant S1-Fc fusion proteins formulated with either CFA & AD11.10 (CFA was used to prime the primate at the first injection and AD11.10, a saponin based microemulsion, which was used to boost) or AD20Gold^+^ (nanoemulsion with synthetic MPL) were used to immunize mice, rabbits and macaques. Beside high levels of the anti-S1 antibodies elicited, higher neutralizing activities against live SARS-CoV-2 virus and/or pseudo-virus from the anti-sera of macaques and rabbits. Our results demonstrated that the S1-Fc fusion protein could effectively induce humoral immune responses in various animals and, most importantly, can elicit high levels of neutralizing antibodies in non-human primates.

## Author contributions

WLR, RQZ, JXC, JGC, ALH, WG contributed the development of recombinant proteins. FF, STG, JCS, YLH, GZ, YXC, XJ, XC, ML, CXX contributed in the antibody generation and sample testing. GFG, BW, JX, GG helped in experiment designs. HS, SS, QW contributed in technical writing.

## Declaration of Competing Interest

The authors declare that they are affiliated with up to two of four commercial entities with stakes in the data.
