# Supplementary material for: Recombinant SARS-CoV-2 spike S1-Fc fusion protein induced high levels of neutralizing responses in nonhuman primates
Source: Vaccine. 2020 Jul 31;38(35):5653–8. doi: 10.1016/j.vaccine.2020.06.066 (PMC7311893; doi:10.1016/j.vaccine.2020.06.066)
Supplement: Supplementary data 1 [file mmc1.docx]

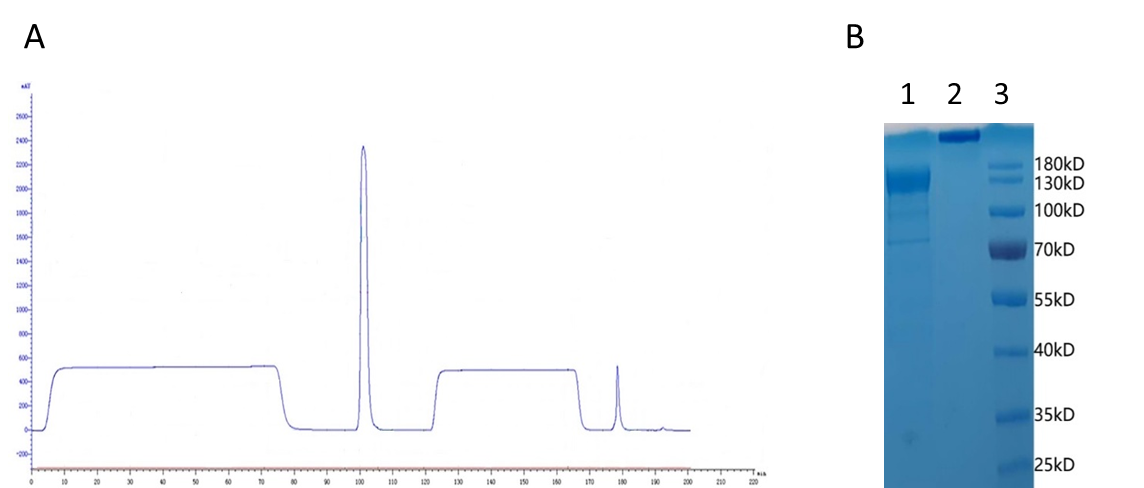


**Fig.S1 Purification of SARS-CoV-2 S1-Fc fusion protein.**

S1-Fc fusion protein was purified from the culture supernatant using Protein A column. A) The horizontal coordinate is time and the vertical coordinate is absorbance value at 280nm. B) SDS-PAGE analysis of purified S1-Fc fusion protein. Lane1, reduced S1-Fc protein; Lane 2, nonreduced S1-Fc; Lane 3 PageRuler™ Prestained Protein Ladder.

**Table S1 Lise of the animal immunization processes**

| Animal | Adjuvant | Dose | Volume | Administration | Time |
| --- | --- | --- | --- | --- | --- |
| Mouse | AD20Gold+ | 9.2 μg | 0.3 mL | I.M. | Day 0 |
|  | AD20Gold+ | 9.2 μg | 0.3 mL | I.M. | Day 3 |
|  | AD20Gold+ | 9.2 μg | 0.3 mL | I.M. | Day 7 |
|  | AD20Gold+ | 0.575 μg | 0.3 mL | I.M. | Day 9 |
|  | AD20Gold+ | 0.575 μg | 0.3 mL | I.M. | Day 11 |
| Rabbit | AD20Gold+ | 100 μg | 0.5 mL | I.M. | Day 0 |
|  | AD20Gold+ | 100 μg | 0.5 mL | I.M. | Day 4 |
|  | AD20Gold+ | 100 μg | 0.5 mL | I.M. | Day 7 |
|  | AD20Gold+ | 50 μg | 0.5 mL | I.M. | Day 11 |
|  | AD20Gold+ | 50 μg | 0.5 mL | I.M. | Day 14 |
|  | AD20Gold+ | 50 μg | 0.5 mL | I.M. | Day 18 |
| Macaque | CFA | 250 μg | 0.5 mL | S.C. | Day 0 |
|  | AD20Gold+ | 250 μg | 0.5 mL | I.M. | Day 4 |
|  | AD20Gold+ | 250 μg | 0.5 mL | I.M. | Day 9 |
|  | AD20Gold+ | 250 μg | 0.5 mL | I.M. | Day 22 |
|  | AD20Gold+ | 250 μg | 0.5 mL | I.M. | Day 26 |

Note: S.C., Subcutaneous; I.M., intramuscular.
